# Fontan Surgical Planning: Numerical Simulations Reveal Efficient Geometries Predicting Post-Surgical Outcomes

**DOI:** 10.21470/1678-9741-2024-0217

**Published:** 2025-03-13

**Authors:** Paulo Cesar Duarte Junior, Alexandre Noboru Murakami, Rudolf Huebner, Hemerson Donizete Pinheiro

**Affiliations:** 1 Department of Bioengineering, Instituto Dante Pazzanese de Cardiologia, São Paulo, São Paulo, Brazil; 2 Department of Clinical Surgery, Faculdade de Medicina da Universidade Estadual de Londrina (UEL), Londrina, Paraná, Brazil; 3 Department of Mechanical Engineering, Universidade Federal de Minas Gerais, Belo Horizonte, Minas Gerais, Brazil; 4 Department of Civil Construction, Universidade Estadual de Londrina, Londrina, Paraná, Brazil

**Keywords:** Hydrodynamics, Pulsative Flow, Pulonary Artery, Pathologic Constriction, Surgical Anastomosis, Right Heart Bypass, Surgeons

## Abstract

**Introduction:**

Computational fluid dynamics has the potential to assist cardiovascular surgeons in making more accurate decisions, allowing the prediction of post-surgical outcomes, provided that pre-surgical conditions are well established. However, the application of current techniques, which are based on volume methods, is still limited to a few specialized centers. Lack of knowledge, coupled with the need for advanced computational resources, can serve as obstacles to implementation.

**Objective:**

This study aimed to develop a replicable surgical planning procedure for a simplified and clinically feasible total cavopulmonary geometry.

**Methods:**

The finite volume method was used to simulate different configurations of cavopulmonary anastomosis under continuous and pulsatile flow and thus gain a better understanding of blood behavior, energy efficiency, and shear stress in the studied regions.

**Results:**

Two geometries were found to be efficient in distributing blood flow in a physiological manner, with adequate shear stress and energy loss. In addition to the correct placement of the anastomosis, the results underscored the need for attention regarding potential stenoses in pulmonary arteries to obtain adequate geometries.

**Conclusion:**

The developed method proved to be effective for early visualization of post-surgical results, particularly in complex clinical cases. Furthermore, the method contributes to a comprehensive understanding of hemodynamics in the studied area, improving the accuracy of cardiovascular surgical planning.

## INTRODUCTION

For every 2,500 children born, one is diagnosed as having only one single functional ventricle (SV)^[1]^. Elective palliative treatment consists of a three-stage surgical procedure culminating in Fontan surgery^[2,3]^. Completion of surgical procedures results in the formation of a total cavopulmonary connection (TCPC), allowing blood to flow directly from the venae cavae to the pulmonary arteries in an SV pumping system^[4,5]^. The high complexity of this staged treatment approach, which entails significant alterations in blood flow during each surgery, represents a challenge to pediatric cardiac surgeons^[6]^. Procedures frequently prove unsuccessful, leading to a survival rate to adulthood of < 50%^[7,8]^. Some reasons for this high rate of failure include the uneven distribution of blood flow from the inferior vena cava (IVC) and superior venae cava (SVC) to the right and left pulmonary arteries, occurrence of non-physiological shear stresses on the vessel wall, and elevated energy loss within the final circuit^[9]^.

The high variability in anatomy and boundary conditions among patients with SV makes it simplistic and dangerous to generalize TCPC parameters, such as anastomosis location, graft diameter, and fenestration geometry^[9,10]^. Each case is unique and requires a tailored approach encompassing multiple variables, such as the patient's clinical history, vascular anatomy, cardiac function, and presence of associated anomalies^[11]^. It is essential that the multidisciplinary team adopts a holistic and individualized perspective based on an in-depth evaluation of each case.

**Fig. 1:**
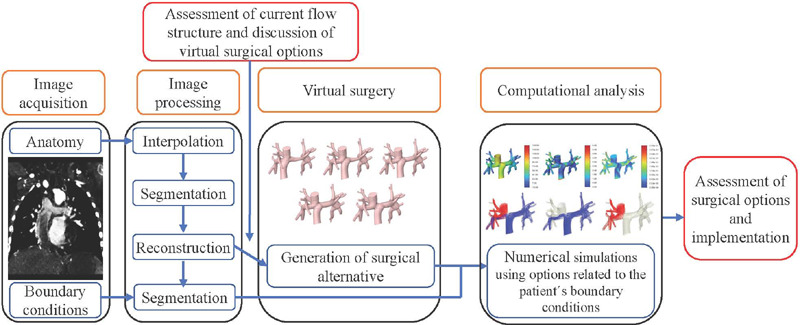
Steps in Fontan surgical planning.

In the 2000s, with the aim of achieving this level of treatment customization, doctors and engineers led a collaborative effort between several areas of science to develop a pre-surgical planning method known as Fontan surgical planning (FSP)^[11,12]^. FSP provides a deep understanding of possible complications and recommends strategies to prevent them, enabling customized simulation of TCPC geometries and hemodynamics and facilitating the identification of the optimum surgical design for each patient^[10]^. FSP is divided into four basic steps and two evaluation steps, as illustrated in Figure 1. First, images of the patient's anatomy are acquired by computed tomography or magnetic resonance imaging. Subsequently, these images need to be treated for use in computational modeling^[9,10]^.

Information is also obtained on the blood flow in the IVC and SVC, pulmonary arteries, potential fenestration, aortic artery, pulmonary veins, and other vessels of interest. A multidisciplinary team composed of engineers, cardiologists, and surgeons perform the virtual surgery and, through computational fluid dynamics (CFD), assess the physiology and hemodynamics of each alternative. Finally, the team determines the best surgical option and proceeds with the surgery^[9]^.

The main objectives of FSP are to minimize energy loss in the TCPC and to ensure a balanced distribution of hepatic flow to the left and right lungs, thereby avoiding excessive shear stress on the vessel wall. Energy loss has been associated with exercise intolerance and is believed to influence the patient's overall prognosis. On the other hand, uneven distribution of hepatic flow is associated with high shear stress on the vessel wall and the development of pulmonary arteriovenous malformation^[9,10]^.

Despite its remarkable benefits, the use of FSP is not widespread. Several factors explain the low adherence to this technique, such as the need for substantial computational resources, lack of specialized multidisciplinary teams, scarcity of research and teaching centers, and lack of funding^[6,13]^. In an effort to reduce the time required for convergence of numerical results and the demand for computational resources, Duarte Junior et al.^[10]^ (2024) investigated the importance of different variables to result accuracy. The authors identified two sets of variables as crucial under different circumstances. Newtonian fluid, turbulent flow, and steady regime in rigid walls were found essential in predicting the distribution of blood flow to the pulmonary arteries. On the other hand, Newtonian fluid, turbulent flow, and transient regime in rigid walls were found to be essential variables when the distribution of blood flow to the pulmonary arteries, shear stress, and energy loss were equally relevant to the predictions. In the first set of parameters, the fluid viscosity is assumed to be constant and independent of the deformation rate. This model considers chaotic fluctuations in flow common to large blood vessels and assumes that flow conditions do not vary with time, while disregarding the deformability of vascular walls to simplify calculations. In the second set of parameters, in addition to maintaining the viscosity constant and independent of the deformation rate and assuming chaotic fluctuations in blood flow, flow conditions are allowed to vary over time. The second model also disregards vessel deformation for simplification purposes.

The frequent incidence of SV and the inherent challenges in its surgical treatment are complex issues requiring an individualized, comprehensive approach from health professionals. In view of the foregoing, this study aimed to apply this approach in a simplified and clinically viable CFD, considering two sets of variables, one for steady flow and the other for complex, pulsatile flow.

## METHODS

Three efficiency parameters should be considered in FSP^[14]^:

Distribution of blood flow between the lungs: the rate of blood flow of the right pulmonary artery to the left pulmonary artery (RPA/LPA) should be in the range of 60/40 to 40/60, with 50/50 being the optimal value;
IVC wall shear stress: the physiological range of wall shear stress in large veins is 0.1–1 Pa. Shear stress values < 0.1 Pa are below the physiological range; and
Lowest possible loss of energy during the passage of fluid through TCPC.

In this study, both the analysis of the distribution of blood flow from the venae cavae to the pulmonary arteries and assessment of wall shear stress were performed through simulations based on CFD, executed using Ansys® software version 2022R^[15]^. Flow equations were solved using the finite volume method. Boundary conditions were included in the general transport equation for comparison and interpretation of the results.

Dissipated energy (E_diss_) was determined from the difference between input energy (E_in_) and output energy (E_out_), expressed by equations (1) and (2), respectively^[16]^:


1) $$E_{in}=Q_{IVC}\left(P_{IVC}+\frac{1}{2}\rho V_{IVC}^{2}\right)+Q_{SVC}\left(P_{SVC}+\frac{1}{2}\rho V_{SVC}^{2}\right)$$

2) $$E_{out}=\sum_{i=1}^{N_{L}}Q_{LPA_{i}}\left(P_{LPA_{i}}+\frac{1}{2}\rho V_{LPA_{i}}^{2}\right)+\sum_{i=1}^{N_{R}}Q_{RPA_{i}}\left(P_{RPA_{I}}+\frac{1}{2}\rho V_{RPA_{i}}^{2}\right)$$

where *N*_R_ is the number of RPA outlets, *N*_L_ is the number of LPA outlets, *V* and are the velocity and pressure averaged over each inlet and outlet face, Q is the flow rate over each face of the model, ρ the density of blood (1060 kg/m^3^).

The smaller the Edi_ss_, expressed by equation (3), the better the geometry. 


3) $$E_{diss}=E_{in}-E_{out}$$

**Fig. 2:**
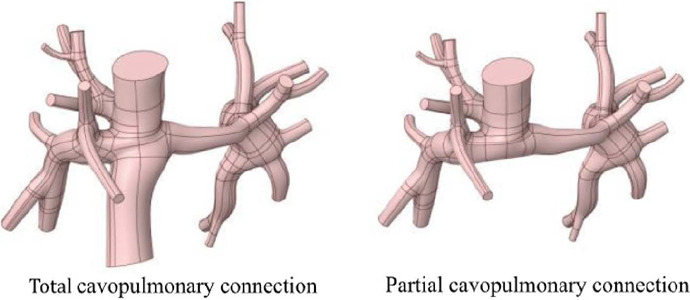
Sectioning of the inferior vena cava of patient B.

FSP was performed using the geometry of Patient 0064, obtained in STL format from a database^[17]^. The six-year-old female patient had undergone TCPC for the treatment of hypoplastic left heart syndrome. As the first step in FSP is determining the geometry for partial cavopulmonary connection (PCPC), it was necessary to alter the original geometry and remove the IVC. This alteration was performed using Ansys SpaceClaim® software version 2022R^[15]^ (Figure 2).

**Fig. 3:**
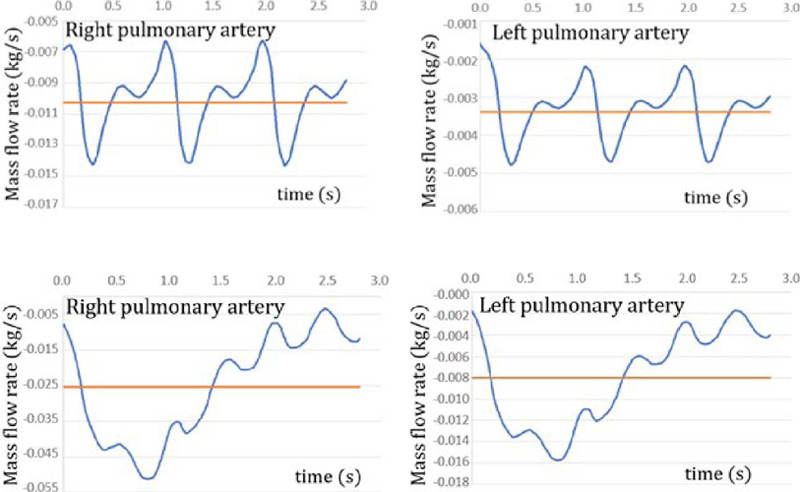
Pulsatile flow profile at rest, transient flow rates, and average flow rate.

With the geometries collected, the next step was to transform them into solids and polish the data. Meshes were successfully generated for PCPC and TCPC geometries, achieving satisfactory quality, as detailed in Appendix A. All the meshes generated in the study were predominantly composed of tetrahedral elements and contained five layers of prismatic elements on the domain surface. Initially, simulations were performed for both geometries in steady regime (continuous blood flow) and transient regime (pulsatile blood flow). All simulations considered vessels as rigid, the fluid as Newtonian, and the behavior of blood flow as turbulent. The shear stress transport k-ω turbulence model was used, as it delivers good precision at a relatively low computational cost^[10,18,19]^. The blood density ρ was set at 1060 kg/m^3^ and the dynamic viscosity (µ) at 4 × 10^−3^ Pa.·s^[2,3,20,21]^. Patient data, including body surface area, IVC pressure, SVC pressure, left pulmonary artery pressure, right pulmonary artery pressure, and cardiac index, are described in Table 1^[15,17]^. Using pressure and flow data, along with vessel cross-sectional areas, it was possible to calculate the blood velocity in IVC and SVC (Table 1). The literature provides reliable information on the distribution of blood flow between IVC and SVC, indicating that, in patients at rest, 60% of the cardiac output flows to IVC, whereas the remaining 40% flows to SVC^[17]^. These percentages were adopted to simulate flow distribution in this study (Table 1). The pulsatile flow profiles were obtained by shifting the curves constructed by Bazilevs et al.^[3]^ (2009). This procedure was undertaken to ensure precise alignment of the average values of curves with velocities reported in the literature^[10,17,22]^. For simulation of the PCPC geometry, the flow rates were set at 0.003 kg/s for LPA and 0.010 kg/s for RPA. For the original TCPC geometry, the flow rates were assumed as 0.008 kg/s for LPA and 0.025 kg/s for RPA (Figure 3).

**Appendix A:** Number of nodes and elements of geometries and quality indices.

Geometry	Nodes	Elements	Skewness			Standard deviation	Orthogonal quality			Standard deviation
			Minimum	Maximum	Mean		Minimum	Maximum	Mean	
TCPC	292,052	835,7	3.50E-04	0.84445	0.22578	0.12342	0.15555	0.99226	0.77326	0.12232
PCPC	252,392	707,16	4.60E-04	0.84182	0.22791	0.12451	0.15818	0.99131	0.77117	0.12345
F_10mm_ with 8 mm LPA	283,66	814,232	2.70E-04	0.84113	0.22589	0.12316	0.15887	0.99375	0.77315	0.12203
F_10mm_ with 10 mm LPA	287,344	827,594	2.50E-04	0.84568	0.22446	0.12229	0.15432	0.99447	0.77459	0.12117
F_15mm_ with 10 mm LPA	287,647	828,999	2.92E-04	0.84987	0.22403	0.12306	0.15013	0.99217	0.77499	0.12193

LPA=left pulmonary artery; PCPC=partial cavopulmonary connection; TCPC=total cavopulmonary connection

**Table 1:** Patient’s characteristics used for determination of boundary conditions^[16,19]^.

**Patient code**	**BSA (m^2^)**	**IVC*_P_* (mmHg)**	**SVC*_P_* (mmHg)**	**LPA*_P_* (mmHg)**	**RPA*_P_* (mmHg)**	**CI (L/min/m^2^)**	***Q* (m^3^/s)**	**QIVC (m^3^/s)**
64	0.71	9	9	6	6	2.7	3.2E-05	1.92E-05

***Q*SVC (m^3^/s)**	**IVC area (m^2^)**	**SVC area (m^2^)**	**Mean IVC diameter (m)**	**Mean SVC diameter (m)**		***V*IVC (m/s)**		***V*SVC (m/s)**
1.28E-05	1.63E-04	1.63E-04	1.44e-02	1.44E-02		0.1175		0.0783

BSA=body surface area; CI=cardiac index; IVC=inferior vena cava; LPA=left pulmonary artery; *_P_*=pressure; *Q*=flow rate; RPA=right pulmonary artery; SVC=superior vena cava; *V*=velocity

**Fig. 4:**
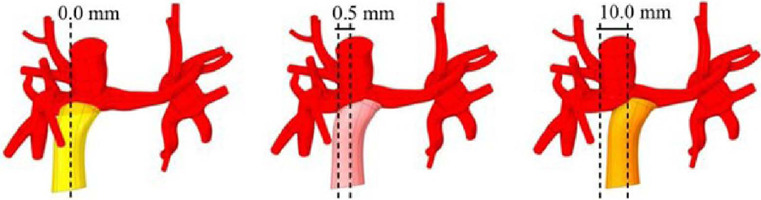
Planning of the placement of the inferior vena cava.

Convergence of the simulations using Ansys® software 2022R^[15]^ revealed that 75% of the blood flow was directed to RPA and 25% to LPA in both geometries (PCPC and TCPC). In view of this result, two new geometries were created in an attempt to shift the blood flow toward LPA, one by displacing the IVC by 5 mm toward the LPA (F_5mm_) and the other by displacing the IVC by 10 mm (F_10mm_) (Figure 4).

**Fig. 5:**
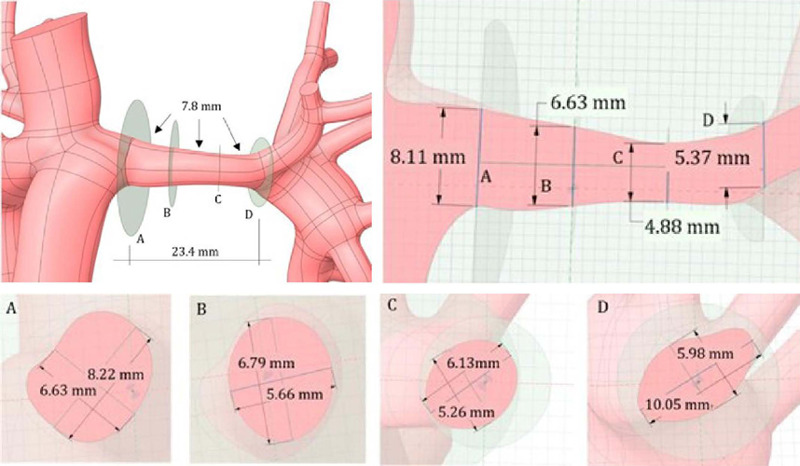
Measurement of left pulmonary artery dimensions.

Achieving an even distribution of flow between pulmonary arteries was the primary goal. At this stage, simulations were performed for a continuous regime, given the increased reliability of this boundary condition^[10,16,22]^. Simulation convergence showed flow distributions of **75.43%** to RPA and **24.12%** to LPA in the F_5mm_ geometry and **75.73%** for RPA and **24.27%** for LPA in the F_10mm_ geometry. That is, the displacement strategies resulted in no significant changes compared with the original TCPC flow. This finding is likely attributable to stenosis at LPA. Therefore, it was necessary to intervene in this region to dilate the stenosis. Only then would it be possible to determine the best placement for IVC. First, LPA was sectioned close to the IVC (Figure 5A) and at the base of the first lobes (Figure 5D). The LPA length was 23.4 mm. It was then decided to take measurements of two points about 7.8 mm apart (23.4/3 mm), which required artery segmentation, as illustrated in Figure 5.

**Fig. 6:**
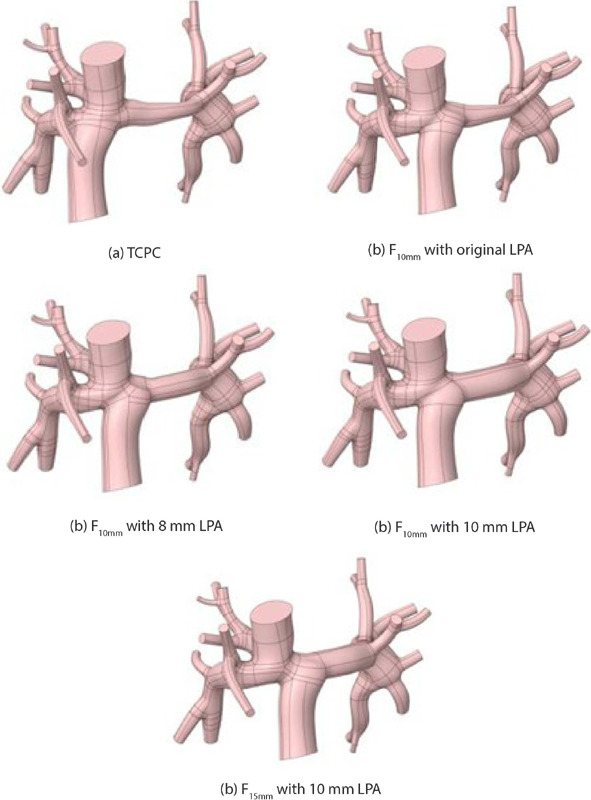
Geometries analyzed in Fontan surgical planning. LPA=left pulmonary artery; TCPC=total cavopulmonary connection.

The average RPA diameter near the anastomosis of the SVC was used for determination of optimum LPA measurements. The average difference in cross-sectional area between LPA and RPA was 40.52%. On the basis of these results, the LPA geometry was initially adjusted to an average diameter of 8 mm. For investigation of the effects of LPA dilation, a geometry with an average LPA diameter of 10 mm was also constructed. Thus, a set of geometries was used to determine whether the desired hemodynamics could be achieved (Figure 6). In addition to the TCPC geometry and the F_10mm_ geometry with the original LPA, three additional geometries were generated, as follows: F_10mm_ with 8 mm LPA (IVC shifted to the left by 10 mm and LPA dilated to 8 mm), F10mm with 10 mm LPA (IVC shifted to the left by 10 mm and LPA dilated to 10 mm), and F_15mm_ with 10 mm LPA (IVC shifted to the left by 15 mm and LPA dilated to 10 mm).

## RESULTS

All meshes were successfully generated with an adequate number of nodes and elements, as well as satisfactory quality parameters (Appendix A). The results were organized into original and modified geometries. Table 2A presents the results for the following simulation conditions: PCPC_steady_, PCPC geometry, rigid vessels, Newtonian fluid, turbulent flow, and steady regime; PCPC_transient_, PCPC geometry, rigid vessels, Newtonian fluid, turbulent flow, and transient regime; TCPC_steady_, original TCPC geometry, rigid vessels, Newtonian fluid, turbulent flow, and steady regime; TCPC_transient_, original TCPC geometry, rigid vessels, Newtonian fluid, turbulent flow, and transient regime. Table 2B shows the results for simulations of modified TCPC geometries in steady regime.

**Table 2:** Results of Fontan surgical planning simulations.

**(a) Code**	**∆*P* (Pa)**	***Q*RPA**	***Q*LPA**	**Mass balance**	**Mean wall shear stress (Pa)**	
					***IVC***	***Geometry***
PCPC_steady_	32.97	74.99%	25.01%	4.8E-08	****	0.53
PCPC_transient_	34.74	75.21%	24.79%	3.2E-08	****	0.54
TCPC_steady_	130.02	75.61%	24.39%	4.4E-08	0.93	1.64
TCPC_transient_	164.82	75.98%	24.02%	3.8E-06	1.19	1.86

**(b) Code (steady regime)**	**∆*P* (Pa)**	***Q*RPA**	***Q*LPA**	**Mass balance**	**Mean wall shear stress (Pa)**	
					***IVC***	***Geometry***
TCPC_steady_	130.02	75.61%	24.39%	4.4E-08	0.93	1.64
F_10mm_ with original LPA	134.93	75.73%	24.27%	1.9E-07	0.94	1.65
F_10mm_ with 8 mm LPA	93.01	64.91%	35.09%	2.7E-07	0.92	1.43
F_10mm_ with 10 mm LPA	78.42	57.09%	42.91%	4.1E-06	0.91	1.31
F_15mm_ with 10 mm LPA	75.61	56.17%	43.83%	1.7E-08	0.87	1.31

IVC=inferior vena cava; LPA=left pulmonary artery; *P*=pressure; PCPC=partial cavopulmonary connection; *Q*=flow rate; RPA=right pulmonary artery; TCPC=total cavopulmonary connection ****Non-existent

**Fig. 7:**
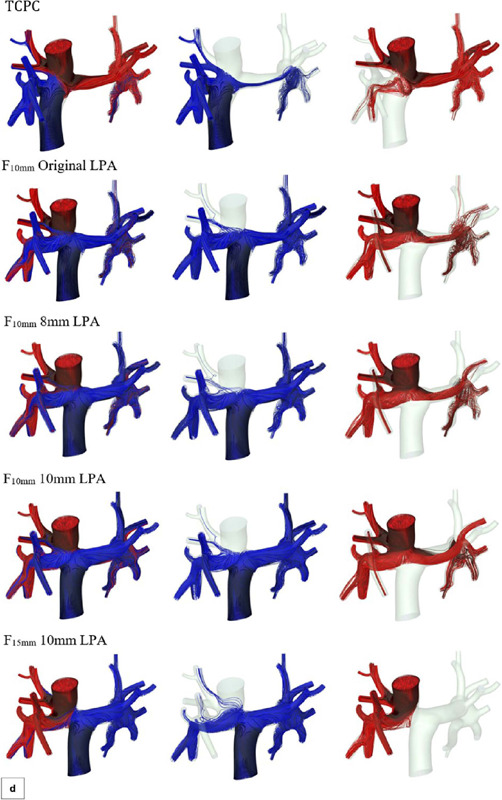
Qualitative results of Fontan surgical planning (a) Static pressure (Pa) (b) Wall shear stress (Pa) (c) Velocity (m/s) (d) Influence of the mean flow from venae cavae to pulmonary arteries LPA=Ieft pulmonary artery; TCPC=total cavopulmonary connection

In addition to numerical results, qualitative variables were also extracted from continuous simulations, as illustrated in Figure 7.

**Figure:**
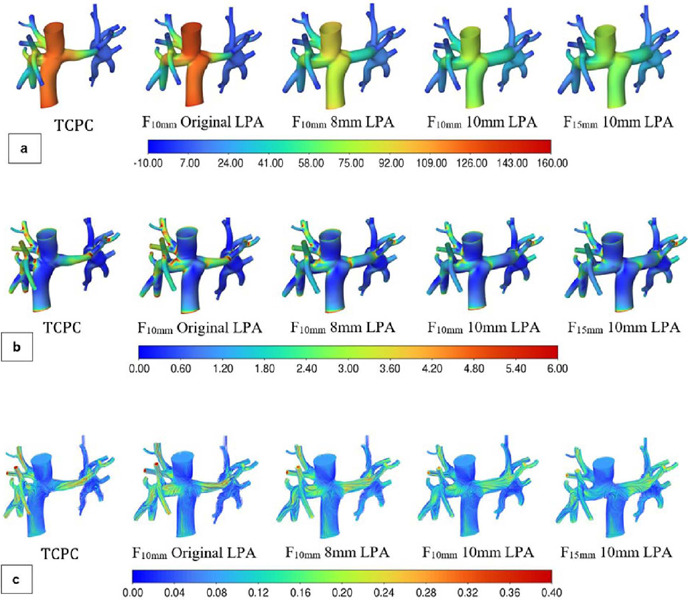

## DISCUSSION

PCPC and TCPC simulations for steady and transient regimes differed significantly in ∆P (difference of 5.35% between PCPC_steady_ and PCPC_transient_ and of 26.76% between TCPC_steady_ and TCPC_transient_) and shear stress (difference of 2.75% between PCPC_steady_ and PCPC_transient_ and of 13.86% between TCPCs and TCPC_transient_) (Table 2A). RPA and LPA flow rate values were similar for all simulations.

In transient simulations, shear stress averages were calculated over one respiratory cycle. In the PCPC simulation, which considers only the inflow through the SVC, it was observed that the variation in mean shear stress is synchronized with the cardiac cycle.This finding suggests a direct influence of cardiac activity on the dynamics of blood flow. On the other hand, in the TCPC simulation, the pattern more closely resembled that of the influence of respiration on blood flow in the IVC. Thus, the variation in shear stress seems to be more affected by respiratory dynamics than by cardiac activity. These results highlight the importance of considering not only vascular anatomy but also the complex interaction between the cardiovascular and respiratory systems when studying blood flow patterns in physiological and pathological conditions.

In the TCPC case, which exhibited vessel stenosis, flow distribution in the pulmonary arteries improved significantly when considering an 8 mm LPA in the F_10mm_ geometry. Without dilation (F_10mm_ with original LPA), the RPA/LPA flow distribution was 75.73/24.27, changing to 64.91/35.09. The pressures in the cavae reduced by 31%, shear stress of the IVC walls decreased by 2%, and that in the rest of the geometry reduced by 13%. Nevertheless, it would be necessary to achieve an RPA/LPA ratio ranging from 60/40 to 40/60. For the F_10mm_ geometry with a 10 mm LPA, flow distribution through the pulmonary arteries improved even more, changing from 64.91/35.09 RPA/LPA in the F_10mm_ with 8 mm LPA to 57.09/42.91. Pressures in the cavae reduced by an average of 16%, shear stress on the IVC wall by 1%, and shear stresses on the rest of the geometry by 8%. Although this geometry met the necessary requirements, an additional simulation was performed. In this simulation, the graft was displaced by 15 mm and the LPA was dilated to 10 mm (F_15mm_ with 10 mm LPA). In this scenario, the final RPA/LPA flow distribution was 56.17/43.83. The pressures in the cavae reduced by 3.59% and shear stress on the IVC wall by 4.64%. The results of all simulations are depicted in Table 2.

The simulation revealed a limit to the increase in LPA diameter, attributed to restrictions in the region of lobe origin. Further dilation would be unfeasible. Graft displacement, although feasible, would necessitate an adequate surgical field. It is interesting to note that vessel dilation resulted in a reduction in ∆P and the mean shear stress on the vessel wall, which is an expected effect. An efficient geometry is characterized by homogeneous flow through the pulmonary arteries, physiological levels of shear stress on the graft, and minimal energy loss.

After determining the flow distribution and shear stress of the graft, it was necessary to calculate E_diss_ of each geometry. For this, it is important to consider the relative pressures at each point rather than absolute pressures. Efficiency results are presented in Table 3. Regarding shear stress on IVC walls, in all geometries, some regions had values below the physiological threshold (< 0.1 Pa). Nevertheless, the ranges in shear stress are deemed suitable for the applied technique, which consists of using synthetic grafts. Comparative analysis showed that F_10mm_ with original LPA had inferior performance to the TCPC model. This finding is due to the displacement of IVC over the constrictive vessel, increasing the pressure on the graft and hindering blood flow in the geometry. In general, the results of the other geometries were consistent with the expectations, suggesting a precise application of physical and boundary conditions.

**Table 3:** Efficiency of geometries for Fontan surgical planning in the steady regime.

Order	Simulation	*Q*RPA	*Q*LPA	Mean wall shear stress (Pa)		Dissipated energy
				*IVC*	*Geometry*	
5	F_10mm_ with original LPA	75.73%	24.27%	0.94	1.65	0.0035
4 ↓	TCPC	75.61% ↓	24.39%	0.93 ↓	1.64 ↓	0.0033
3	F_10mm_ with 8mm LPA	64.91%	35.09%	0.92	1.43	0.0024
2	F_10mm_ with 10mm LP	57.09% A	42.91%	0.91	1.31	0.0021
1	F_15mm_ with 10mm LP	56.17% A	43.83%	0.87	1.31	0.0020

IVC=inferior vena cava; LPA=left pulmonary artery; *Q*=flow rate; RPA=right pulmonary artery; TCPC=total cavopulmonary connection

Figure 7A illustrates the decompression in the pressure profile with LPA dilation to 8 mm. This finding is in agreement with quantitative results, which indicated a reduction of 31.07% in ∆P. This gradual reduction continued until the desired geometry was reached (F_15mm_ with 10 mm LPA). Notably, the reduction in shear stress was observed in all qualitative results, being particularly evident in the flow from venae cavae to pulmonary arteries (Figure 7D). Displacement of the IVC by 10 cm and dilation of LPA to 8 mm promoted a more uniform distribution of blood flow (from both SVC and IVC) to the pulmonary arteries (Figure 7D). Differences with the other simulations were not as pronounced for geometries with 10 mm LPA. However, in the last simulation (F_15mm_ 10 mm LPA), the SVC flow was entirely directed to the RPA, whereas the IVC flow fed the LPA, proceeding to the RPA.

After completion of steady regime simulations, a last simulation was performed using F_15mm_ with 10 mm LPA in transient regime. Table 4 provides a comparison of the results for this geometry in steady and transient regimes.

**Table 4:** Results of simulations for Fontan surgical planning in steady and transient regimes.

Simulation	Regime	∆*P* (Pa)	*Q*RPA	*Q*LPA	Mass balance	Mean wall shear stress (Pa)	
						*IVC*	*Geometry*
F_15mm_ with 10 mm LPA	Steady	75.61	56.17%	43.83%	1.7E-08	0.87	1.31
F_15mm_ with 10 mm LPA	Transient	89.37	56.49%	43.51%	5.9E-07	1.05	1.39
*Difference between simulations*		18.20%	2.73%	3.98%		21.58%	5.96%

IVC=inferior vena cava; LPA=left pulmonary artery; *Q*=flow rate; RPA=right pulmonary artery

**Fig. 8:**
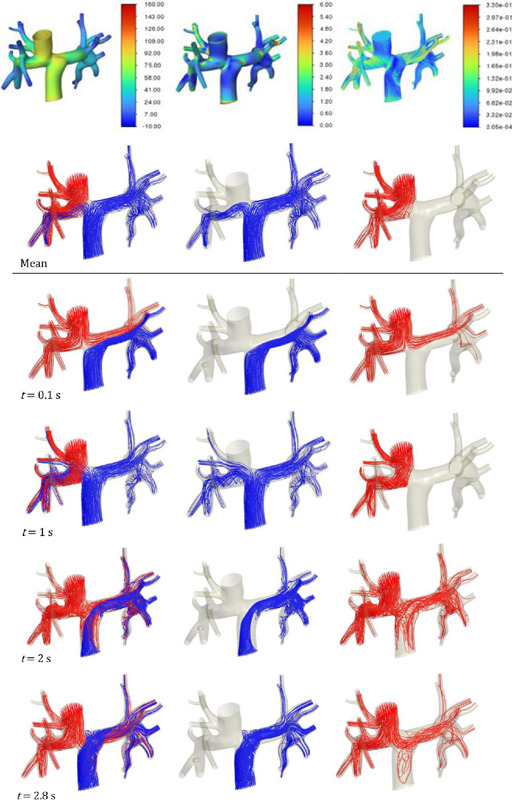
Fontan surgical planning qualitative results for F15m m with 10 mm left pulmonary artery in the transient regime (a)Mean static pressure (Pa) (b) Mean wall shear stress (Pa) (c) Mean velocity (m/ s) (d) Influence of the flow distribution from venae cavae to pulmonary arteries overtime.

The variation in ∆P and wall shear stress, particularly in the IVC region, was evident. Flow rates, by contrast, did not vary much between regimes. The qualitative results for F_15mm_ with 10 mm LPA in transient regime are illustrated in Figure 8. An important aspect of transient simulations is the ability to identify flow distribution in the pulmonary arteries (Figure 8).

IVC flow was partially distributed to the RPA only at one second; at the other times, the flow remained exclusive to LPA. Concomitantly, the SVC flow was exclusive to RPA at one second. At t = 0.1 s, the SVC flow was divided between pulmonary arteries, with greater flow to the RPA. At later times, there was a more equal distribution of the SVC flow among arteries, evidencing the reflux in IVC. The other geometries were not simulated in transient regime, because it was estimated that ∆P and shear stress results would vary proportionally.

### Limitations

Some of the limitations of this study were the costs associated with hardware and software licenses for numerical analysis. We used an Alienware® computer with an Intel® CORE™ i7-11800H processor (8 cores, 24 MB cache, up to 4.6 GHz), NVIDIA® GeForce RTX™ 3070 GPU, 8 GB GDDR6, 64 GB DDR4 memory at 3200 MHz, and two 1 TB PCIe NVMe M.2 SSDs. During processing, the major constraint was found to be due to the processor, even with the use of seven of the eight cores. Most of the time, the processor was used at 100% of its capacity. RAM usage did not surpass 30%, demonstrating that, for the proposed simulations, the processor was the limiting factor for convergence time. Simulations with steady flow required a computational time of about 15 minutes. By contrast, transient simulations required an average processing time of four hours.

## CONCLUSION

FSP using numerical methods is an essential strategy to generate early anatomical models and immediate hemodynamic results with safety and reliability. This study showed that vascular shear stress was improved, and energy loss reduced when blood flow from the venae cavae was better distributed to the pulmonary arteries.

By using simplified parameters, such as Newtonian fluid, turbulent flow, and steady regime in FSP, it was possible to achieve reliable results with less computational effort. Simplification of numerical convergence enhances the accessibility of information to professionals in the field of medical simulation, expanding its applicability in the health sector. This approach facilitates the adoption of advanced surgical planning techniques, contributing to better clinical outcomes and utilization of available resources.

## Abbreviations, Acronyms & Symbols

BSA: Body surface area
CFD: Computational fluid dynamics
CI: Cardiac index
E_diss_: Dissipated energy
E_in_: Input energy
E_out_: Output energy
FSP: Fontan surgical planning
IVC: Inferior vena cava
LPA: Left pulmonary artery
*P*: Pressure
ρ: Density of blood
PCPC: Partial cavopulmonary connection
*Q*: Flow rate
RPA: Right pulmonary artery
SV: Single functional ventricle
SVC: Superior vena cava
TCPC: Total cavopulmonary connection
*V*: Velocity
